# Sustained maternal hyperoxygenation improves aortic arch dimensions in fetuses with coarctation

**DOI:** 10.1038/srep39304

**Published:** 2016-12-16

**Authors:** Shi Zeng, Jiawei Zhou, Qinghai Peng, Wen Deng, Ming Zhang, Yili Zhao, Tao Wang, Qichang Zhou

**Affiliations:** 1 Department of Ultrasonography, The Second Xiangya Hospital, Central South University, Changsha, P.R. China; 2 Department of Gynecology & Obstetrics, The Second Xiangya Hospital, Central South University, Changsha, P.R. China; 3 Department of Pediatric, The Second Xiangya Hospital, Central South University, Changsha, P.R. China

## Abstract

The aim was to investigate the impact of maternal hyperoxygenation (HO) on cardiac dimensions in fetuses with isolated Coarctation (CoA). Fetal echocardiography was performed serially in 48 fetuses with CoA and gestation age matched normal fetues. The Z-scores for the mitral valve (MV), tricuspid valve (TV), aortic valve (AV), ascending aorta (AAo), isthmus, pulmonary valve (PV), main pulmonary artery (MPA), and descending aorta (DAo) were measured and compared among normal fetuses, CoA fetuses with oxygen and CoA fetuses with air. In the group with oxygen, 6 L/min oxygen was administered to the mother using a face mask. Regression analyses were performed to identify potential factors for HO outcome. The left heart dimension Z-scores increased gradually during HO therapy periods, especially at 4 weeks after oxygen therapy (P < 0.05). As for the case group with air, the left heart dimension remained unchanged. The duration of HO was associated with aortic arch Z-scores (adjusted R^2^ = 0.199, 0.60 for AAO and isthmus, respectively). Sustained maternal middle-flow oxygenation can be safely used to improve left heart dimensions in fetuses with isolated CoA. The duration of HO were associated with treatment outcome. These findings may provide useful information for developing novel treatment strategies.

Coarctation of the aorta (CoA) is the seventh most common congenital heart defect (CHD), accounting for 6–8% of all live births with CHD^1^,^2^. CoA may affect the aorta in several ways, from a discrete constriction of the aortic isthmus to the more extreme scenario of severe tubular hypoplasia. Antenatal diagnosis of aortic coarctation can result in reduced mortality and improved preoperative hemodynamic stability by maintaining duct patency^3^. Although there is a high false-positive diagnostic rate in detecting CoA prenatally, recent reports emphasized the Z-scores of isthmal diameter with abnormal isthmus flow as sensitive and specific indicators of fetal coarctation^4^,^5^,^6^.

Due to significant advances in technique, transcatheter intervention and surgical approaches for CoA have extremely low mortality and infrequent morbidity in the short term. However, the rate of late cardiovascular mortality is as high as 18%, and longer term morbidity remains above 50% due to atherosclerotic disease, recoarctation, aneurysm formation, and persistent hypertension^7^,^8^. Sudden death was also reported^9^. Thus, CoA is a life-threatening cardiovascular condition and requires life-long surveillance. Emerging studies suggest that earlier surgery may minimize the risk of late vascular dysfunction^10^. Thus, we hypothesized that intrauterine therapy would have a positive effect in fetuses with CoA.

Materno-fetal hyperoxygenation (HO) therapy has been applied in several conditions, such as intrauterine growth retardation^11^,^12^, pulmonary hypoplasia^13^, and congenital diaphragmatic hernia^14^. Data on fetuses with CHD demonstrated that HO increased fetal pulmonary venous return^15^ and improved hypoplastic cardiovascular dimensions^16^,^17^. We hypothesized that HO therapy can improve cardiac dimensions in fetuses with CoA. A secondary aim of this study was to identify potential factors that may affect oxygen therapy in fetuses with CoA.

## Results

### General condition

A total of 115 fetuses were enrolled. Nine fetuses were excluded for the following reasons: 5 due to excessive fetal motion resulting in poor-quality images, 2 due to pregnancy termination, and 2 due to incomplete postnatal follow-up. In total, 96 fetuses were studied: 48 normal control fetuses and 48 fetuses with Coa (24 fetuses with oxygen and 24 fetuses with air). The clinical characteristics are summarized in Table 1. In the group with oxygen, materno-fetal HO started at a gestational age of 30.44 ± 2.89 weeks (range 26.6–35.3 weeks) and ended at a gestational age of 39.68 ± 0.78 weeks (range 38–41 weeks). HO therapy lasted 9.24 ± 3.16 weeks (range 3.9–14.4 weeks). No baby was found to have an abnormal ocular fundus upon examination, and no mother had an abnormal chest X-ray.

### Time-course change of cardiac dimension

**Figure 1:**
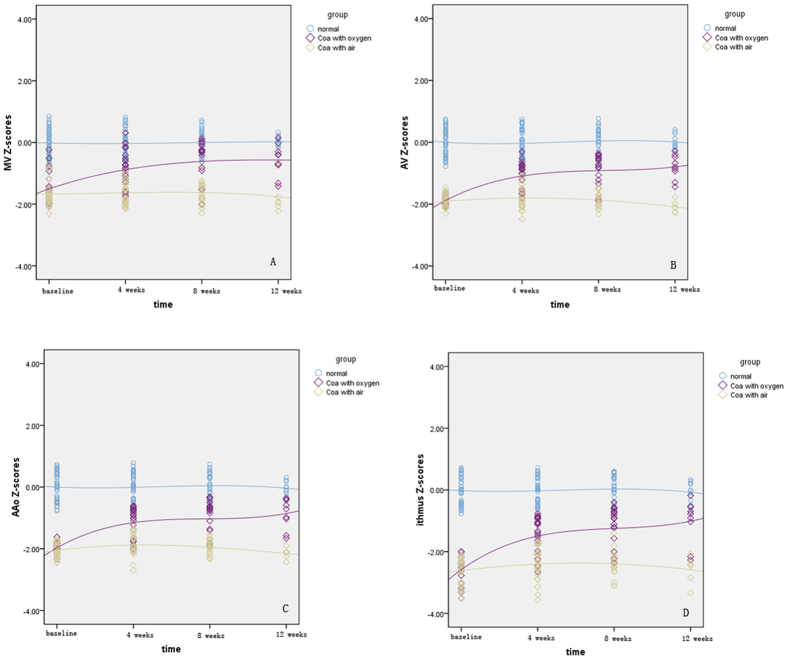
Scatterplots of left heart dimension Z-scores are presented with an interpolation line for the depicted groups. The time-course change of MV Z-scores is demonstrated in (**A**) the change of AV Z-scores is demonstrated in (**B**) the change of AAo Z-scores in (**C**) and isthmus Z-scores in (**D**) MV, mitral valve; AV, aortic valve; AAo, ascending aorta.

At the baseline, the left heart dimension Z-scores in both cases groups were smaller than those in normal fetuses. As for the case group with oxygen, the left heart dimension Z-scores increased gradually during HO therapy periods (P < 0.01), especially at 4 weeks after oxygen therapy. The left cardiac dimension grew near to normal level at the end of HO therapy. Additionally, compared with the isthmus flow pattern at baseline, fewer fetuses exhibiting abnormal flow at the end of oxygen therapy [24/24 (100%) VS 5/24 (20.8%), P < 0.01]. However, as for the case group with air, the left heart dimension Z-scores remained unchanged (P > 0.05) and were smaller than those in control group (P < 0.01) (Fig. 1).

**Figure 2:**
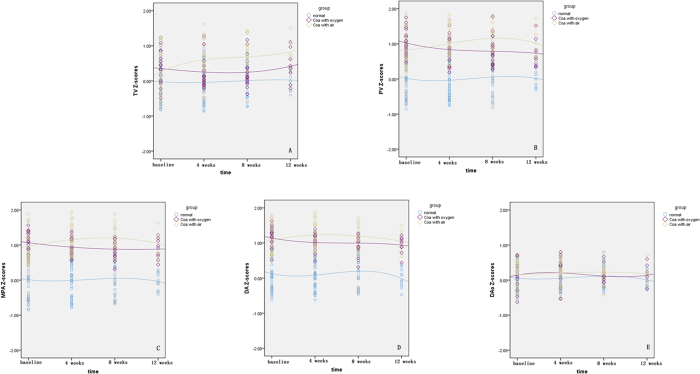
Scatterplots of right heart dimension Z-scores are presented with an interpolation line for the depicted groups. The time-course change of TV Z-scores is demonstrated in (**A**) the change of PV Z-scores is demonstrated in (**B**) the change of MPA Z-scores in (**C**) DA Z-scores in (**D**) and DAo Z-scores in (**E**) TV, tricuspid valve; PV, pulmonary artery valve; MPA, main pulmonary artery; DA, arterial duct; DAo, descending aorta.

The right cardiac dimension Z-scores in three groups remained unchanged during serial scans (P > 0.05) (Fig. 2).

### HO efficacy group VS inefficacy group

At the end of HO therapy, the participants in the group with oxygen were divided into the efficacy group if the isthmus Z-score ≥ −2 and the inefficacy group if the isthmus dimension Z-score < −2. As a result, the efficacy and inefficacy groups contained 18 and 6 fetuses, respectively. Among the eighteen fetuses in the efficacy group, 17 were considered normal after duct closure and one was kept under surveillance. Among the 6 fetuses in the inefficacy group, 5 underwent surgery and 1 was kept under surveillance. There were more patients complicated bicuspid aortic valve [4/6 (66.7%) VS 2/18 (11.1%), P < 0.05], and aortic valve stenosis [3/6 (50%) VS 1/6 (5.5%), P < 0.05] in inefficacy group when compared with efficacy group. There were no significant difference of treatment duration and maternal arterial PO_2_ between the two group. (Table 2).

**Figure 3:**
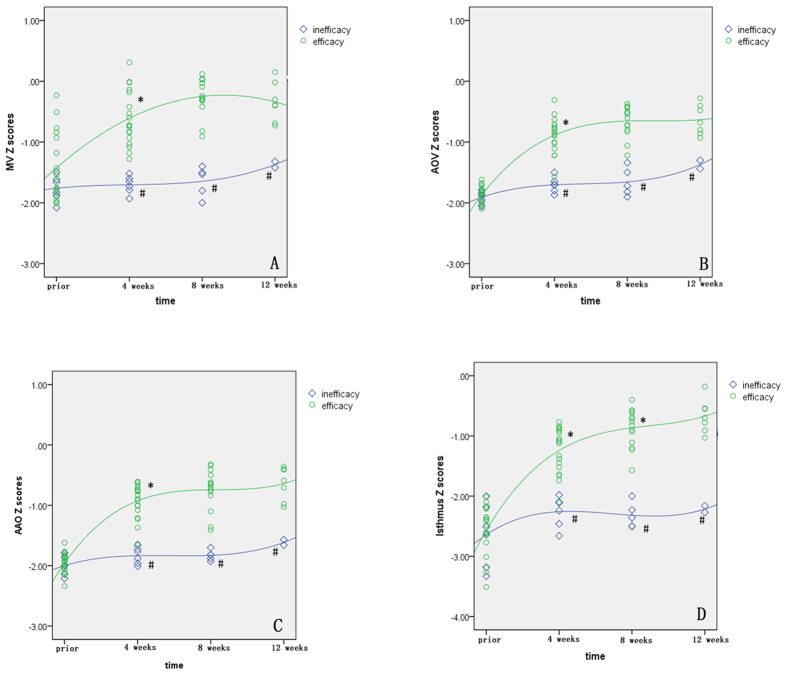
Scatterplots of left heart dimension Z-scores are presented with an interpolation line for the depicted groups. (**A**) Demonstrates the relationship between MV Z-scores and HO therapy. (**B**) Demonstrates the relationship between AOV Z-scores and HO therapy. (**C**) Demonstrates the relationship between AAO Z-scores and HO therapy. (**D**) Demonstrates the relationship between isthmus Z-scores and HO therapy. MV, mitral valve; AOV, aortic valve; AAO, ascending aorta. *P < 0.05 compared with the earlier time point; ^#^P < 0.05 compared with the efficacy group at the same time point.

In the efficacy group, the LV dimension increased as a function of time as follows: at 4 weeks, Z-scores for MV, AOV, AAO, and isthmus dimensions were significantly greater than those obtained prior to HO therapy (P < 0.01); at 8 weeks, although the MV, AOV, and AAO dimensions demonstrated an increasing trend that did not reach statistical significance (P = 0.07, 0.06, 0.1, respectively), the Z-scores of the isthmus in the three-vessel and trachea view (3VT) views were significantly greater than those obtained at earlier time points (P < 0.05). At 12 weeks, the LV dimension remained statistically unchanged compared with that at 8 weeks (P > 0.05, Fig. 3). Right heart dimension Z-scores remained unchanged during HO therapy (P > 0.05).

In the inefficacy group, both the left and right heart dimension Z-scores remained unchanged through the HO therapy period (P > 0.05). Compared with the efficacy group, left heart dimension Z-scores were the same prior to HO therapy (P > 0.05) but were significantly smaller at 4, 8, and 12 weeks post-HO therapy (P < 0.05, Fig. 3).

### Impact factors for HO therapy outcome

In the multivariate model, the duration of HO was independently associated with arch dimension Z-scores increase in the efficacy group (adjusted R^2^ = 0.199, P < 0.05 for AAO; adjusted R^2^ = 0.606, P < 0.01 for isthmus).

## Discussion

This study revealed that sustained maternal oxygen therapy could improve left cardiac dimensions especially isthmus dimension in fetuses with coarctation. These findings may provide useful information for the development of novel treatment strategies and promote intrauterine therapy in fetuses with coarctation.

The left cardiac dimension Z-scores were smaller in fetus with CoA than that in normal control fetuses at the baseline. During maternal hyperoxygenation treatment, the Z-scores of the left cardiovascular structures, namely, the MV, AOV, AAO, and isthmus, increased significantly. However, as for the case group with air, the left heart dimension remained unchanged. This result demonstrated that HO therapy may enlarge the left heart dimension to a certain extent in fetuses with CoA. Thomas Kohl^16^ offered materno-fetal HO during the final weeks of gestation in 15 fetuses with varying degrees of hypoplastic cardiovascular structures (13 of the 15 fetuses had ≥1 hypoplastic left heart structures). They found that chronic intermittent HO acutely improved ventricular area, atrioventricular valve diameter, semilunar valve diameter, great arteries and aortic isthmus diameters in most fetuses. Our maternal fetal oxygenation protocol was similar with their: middle flow oxygenation and several hours per day until delivery. In their study duration of treatment was only 8–33 days and primarily after 35 wks GA while HO was administered during the second/third trimester in our study. Moreover, there was an association between HO duration and cardiac dimensions; specifically, the longer the duration of HO, the greater the increase in left heart size. Associated structural cardiac defects or insufficient increase in blood flow through the aorta was thought a factor to lead to a variety of aortic arch abnormalities including coarctation^18^. Chronic pulmonary vasodilation resulting from maternal HO therapy may stimulate the growth of the left-sided structures by increasing LV filling and substantially augmenting anterograde flow in the aortic isthmus. A previous study reported that HO administration resulted in effective pulmonary venous return in both normal fetuses^19^ and fetuses with hypoplastic left heart syndrome^15^.

HO, as a diagnostic or therapeutic tool, has been applied in various prenatal conditions^11^,^12^,^13^,^14^,^15^,^16^,^17^ and generally consists of a short duration of high-flow oxygen inhalation in the third trimester of pregnancy. In this study, chronic intermittent middle-flow oxygen was applied, with half of the participants initially receiving it during the second trimester. Thomas Kohl^16^ reported that ventricular septal defects, as well as obstructions to ventricular filling or emptying, could neutralize the effect of HO. In our study, VSD had no obvious deleterious effects on HO efficacy. This discrepancy may relate to the degree of VSD shunting and case enrollment discrepancies. However, there were more babies complicated with bicuspid aortic valve and aortic valve stenosis in inefficacy group. More interestingly, the only baby requiring monitoring in the efficacy group was confirmed with bifoliate aortic valve and progressive aortic valve stenosis from mild in neonate to modereate at 1 age of year. Therefore bicuspid aortic valve may be a risk factor for oxygenation treatment outcome, while it was hard to be detected in fetus period. Additionally, in the efficacy group, the duration of HO was independently associated with cardiac dimension Z-scores. This result demonstrated that the recoveries of cardiac structure were time-dependent events.

Importantly, middle-flow oxygen administration for months did not cause clinically apparent harm either to babies or mothers during follow-up; we observed no fetuses with ductus arteriosus closure, no babies with an abnormal retina, and no mothers with an abnormal chest X-ray. Retinopathy of prematurity (ROP), initially called retrolental fibroplasia, is characteristic as vasoproliferative retinaopathy and primarily affects neonates born at <32 weeks gestational age. Premature delivery (low gestational age and low birth weight), high oxygen supplementation, poor postnatal weight gain, hyperglycaemia, low IGF-1 concentration, blood transfusion and infection are associated with development of ROP^20^. Although there was no baby complicated with ROP in our study, hyperoxia in utero environment still was a high risk factor contributing to the development of ROP. We divided fetus with oxygen as GA < 32weeks and GA ≥ 32 weeks, and found GA was not associated with the outcome of HO treatment. Considering controversy for oxygen supplemental usage, we recommend that starting O2 at 32 weeks is would be early enough.

This study has several limitations. First, in this pilot study, we recruited the normal fetuses as a “negative” control group and coarctation fetuses without oxygen intervention as a “positive” control group. Although there was no statistically significance on cardiac dimension Z-scores at baseline between the cases with oxygen and without oxygen, we could not sure the hypoplasia degrees of isthmus were the same between the two groups. Moreover, although the number of cases was sufficient to demonstrate statistically significant differences during HO therapy, we did not have the statistical power to predict HO treatment outcomes for each individual. A larger series using a greater number of cases is needed to draw definitive conclusions regarding the clinical usefulness of this new method. As a new therapeutic method, whether this treatment will produce problems in babies and mothers over a longer follow-up period remains to be established.

## Materials and Methods

A prospective investigation was conducted at The Second Xiangya Hospital of Central South University in China. The cases population consisted of pregnant women referred for fetal echocardiography because of ventricular and/or great artery disproportion with a small left heart during the period of January 2012 to December 2015. The inclusion criteria were single fetuses with isolated CoA and disturbed isthmus flow. The diagnostic criterion for CoA is based on the arch size according to Z-score^5^,^6^, namely, a Z-score of the aortic isthmus diameter < −2 when measured just before the descending aorta in the three-vessel and trachea view (3VT). We excluded fetuses with right ventricular apex formation, mitral atresia, aortic atresia, and medium or large ventricular septal defects. Furthermore, fetuses were excluded if they had a gestational age (GA) <18 weeks or >40 weeks, small for gestational age, chromosomal defects, extracardiac abnormalities, or persistent fetal arrhythmia, and they were also excluded in cases of maternal complications, including gestational diabetes, pre-eclampsia, and thyroid disease. The control group consisted of GA matched mothers form local low risk population. This study was approved by the institutional review board at The Second Xiangya Hospital of Central South University and all methods were performed in accordance with the relevant guidelines and regulations. Written informed consent was obtained from all families.

A complete standard fetal echocardiogram was performed in room air using a Voluson E8 (GE Healthcare Ultrasound, Milwaukee, WI, USA) ultrasound system with a RAB 4–8-D curvilinear probe. After confirming the presence of CoA anatomy, written informed consent was obtained from all families. Mothers were randomly divided as group with oxygen and group with air. In the group with oxygen, we administered 45% oxygen (6 L/min) to the mother using a face mask in the mode of 3 hours per morning and per afternoon totally 6 h/d until delivery. All the fetuses were monitored once every 4 weeks by obstetric ultrasound, fetal echocardiography, and cardiotocography, and the mothers were also questioned by an obstetrician. Maternal arterial PO_2_ was measured at the first day and the last day after HO treatment by ultrasound-guided puncture of the maternal femoral artery. After delivery, retinal examinations were performed weekly for babies in the group with oxygen from 4 weeks to 6 weeks after birth using the RetCam digital came, and chest X-rays were performed in all participants in the group with oxygen.

All principal measurements were performed at baseline and repeated every four weeks. For all fetuses, routine obstetrical ultrasound and fetal echocardiography examinations were performed by one expert (Z.QC.). The pregnancy duration was estimated from the day of the last menstrual period and was confirmed by ultrasound measurement during the first trimester. All cardiovascular dimensions were measured from inner edge to inner edge using two-dimensional ultrasonography. The mitral valve (MV) and tricuspid valve (TV) were measured at end-diastole in the four-chamber view. The aortic valve (AV), ascending aorta (AAo), descending aorta (DAo), pulmonary valve (PV), and main pulmonary artery (MPA) were measured during ventricular ejection in the longitudinal view. The aortic isthmus was measured immediately proximal to the insertion of the arterial duct in the 3VT view^21^. The ductal diameter was also measured in the 3VT view. Each dimension was measured three times, and the mean measurement was used to obtain Z-scores using previously published normative data^21^,^22^. In addition, the flow pattern at the isthmus was recorded as normal (forward flow) or abnormal (persistent diastolic blood flow or reverse flow) and demonstrated on either the 3VT or sagittal view^6^ using Doppler with the angle below 10°. Additionally, minor secondary diagnoses of small ventricular septal defects, a bifoliate aortic valve, and a persistent left superior caval vein to the coronary sinus were identified.

The data are reported as the means with SDs or frequencies with percentages, as appropriate. Comparisons for clinical characteristics and serial echocardiogram parameters between controls and fetuses with Coa were performed using Student’s t-tests or χ2 test. The time-course changes in cardiac dimension Z-scores in all groups were assessed using the post-hoc Games-Howell test for multiple comparisons following one-way analysis of variance (ANOVA). Scatterplots are presented with an interpolation line for the depicted groups. The rate of increased arch dimension Z-scores was calculated using the following formula: rate increase = (Z-scores at first time– Z-scores at last time)/the absolute values of Z-scores at baseline. Multiple regression analyses were conducted to identify the factors associated with the rate of increase in the dimensions in the HO group. The independent variables included maternal age, GA at diagnosis, durations of HO, initial Z-score of the isthmus diameter, and presence of a small ventricular septal defect, and left superior vena cava. P < 0.05 was considered to be significant. All statistical analyses were performed using PASW statistics software [PASW (SPSS) statistics, version 18.0, IBM].

## Conclusions

In summary, sustained intermittent maternal middle-flow oxygenation can be safely used to improve left heart dimensions in fetuses with isolated coarctation. The left heart dimension Z-scores increased gradually during HO therapy periods, especially at 4 weeks after oxygen therapy. The duration of oxygenation was associated with cardiac dimension Z-scores. These findings may provide useful information for the development of novel treatment strategies and promote intrauterine therapy in fetuses with CoA.

## Additional Information

**How to cite this article**: Zeng, S. *et al*. Sustained maternal hyperoxygenation improves aortic arch dimensions in fetuses with coarctation. *Sci. Rep.* **6**, 39304; doi: 10.1038/srep39304 (2016).

**Publisher’s note:** Springer Nature remains neutral with regard to jurisdictional claims in published maps and institutional affiliations.

## Figures and Tables

**Table 1:** The clinical characteristics details of the cohort (n = 96).

Characteristics	Control group (n = 48)	Coa fetuses with oxygen (n = 24)	P^∫^	Coa fetuses with air (n = 24)	P^**∫**^	P^**∮**^
Maternal age (years)	26.48 ± 5.12	27.04 ± 5.71	0.91	28.29 ± 5.63	0.38	0.72
GA at diagnosis (weeks)	30.14 ± 2.89	30.14 ± 2.93	1	30.13 ± 2.87	1	1
EFW at diagnosis(g)	1512.71 ± 472.98	1497.67 ± 487.73	0.99	1533.88 ± 479.93	0.98	0.96
Complicated with small VSD(n)	0	7(29.2%)	<0.01	5(20.8%)	<0.01	0.74
with bifoliate aortic valve(n)	0	6(25%)	<0.01	5(20.8%)	<0.01	1
with LSVC (n)	0	7(29.2%)	<0.01	7(29.2%)	<0.01	1
Serial scans four times	11(22.9%)	9(37.5%)	0.27	6(25%)	1	0.53
three times	21(43.8%)	10(41.7%)	1	11(45.8%)	1	1
two times	16(33.3)	5(20.8%)	0.18	7(29.2%)	0.59	0.74
Perinatal outcome cesarean delivery (n)	7(14.6%)	8(33.3%)	0.12	9(37.5%)	<0.05	1
live birth (n)	48(100%)	24(100%)	1	24(100%)	1	1
NND (n)	0	0	1	0	1	1
Postnatal outcome follow up (months)	13.96 ± 9.38	18.00 ± 9.98	0.24	15.46 ± 8.82	0.78	0.62
normal arch (n)*	48(100%)	17(70.8%)	<0.01	3(12.5%)	<0.01	<0.01
surveillance (n)	0	2(8.4%)	0.11	3(12.5%)	<0.05	1
surgery (n)	0	5(20.8%)	<0.01	18(75%)	<0.01	<0.01
baby with abnormal ocular fundus	/	0		/		
mother with abnormal chest X-ray	/	0		/		

GA, gestation age; EFW, Estimated fetal weight; LSVC, persistent left superior caval vein to coronary sinus; HO, Materno-fetal hyperoxygenation; NND, neonatal death. *Normal arch was assessed by echocardiography after the duct closed.

P^∫^, vs normal group; P^∮^, Coa fetuses with oxygen vs Coa fetuses with air.

**Table 2:** The clinical characteristics details between HO efficacy group and HO inefficacy group (n = 24).

	efficacy group (n = 18)	Inefficacy group (n = 6)	P
MV Z score prior HO	−1.42 ± 0.55	−1.76 ± 0.21	0.16
End of HO	−0.38 ± 0.37	−1.62 ± 0.26	P < 0.01
AOV Z score prior HO	−1.87 ± 0.14	−1.91 ± 0.09	0.58
End of HO	−0.67 ± 0.24	−1.65 ± 0.23	P < 0.01
AAO Z score prior HO	−1.96 ± 0.16	−2.00 ± 0.16	0.62
End of HO	−0.72 ± 0.23	−1.79 ± 0.14	P < 0.01
Isthhmus Z score prior HO	−2.57 ± 0.43	−2.64 ± 0.53	0.75
End of HO	−0.94 ± 0.40	−2.36 ± 0.19	P < 0.01
Complicated with small VSD(n)	4(22.2%)	3(50%)	0.31
bifoliate aortic valve(n)	2(11.1%)	4(66.7%)	P < 0.05
Aortic stenosis	1(5.5%)	3(50%)	P < 0.05
Aortic insufficiency	0	1(16.7%)	0.25
LSVC (n)	4(22.2%)	3(50%)	0.31
HO therapy beginning GA (weeks)	30.53 ± 2.86	30.15 ± 3.24	0.78
last period (weeks)	9.24 ± 3.37	9.23 ± 2.69	0.99
ending GA (weeks)	39.78 ± 0.73	39.38 ± 0.89	0.29
Mother PO2(mmHg) the first day of HO	203.38 ± 13.61	202.67 ± 14.19	0.91
the last day of HO	204.06 ± 13.84	201.13±13.85	0.68
Perinatal outcome cesarean delivery (n)	4(34.78%)	4(66.7%)	0.13
live birth (n)	18(100%)	6(100%)	1
NND (n)	0	0	1
Postnatal outcome follow up periods (months)	17.22 ± 9.18	20.33 ± 12.77	0.52
normal arch (n)	17(94.5%)	0	P < 0.01
surveillance (n)	1(5.5%)	1(16.7%)	0.45
surgery(n)	0	5(83.3%)	P < 0.01

GA, gestation age; LSVC, persistent left superior caval vein to coronary sinus; HO, Materno-fetal hyperoxygenation; NND, neonatal death.
